# Determinants of the time-to-peak left ventricular dP/dt (Td) and QRS duration with different fusion strategies in cardiac resynchronization therapy

**DOI:** 10.3389/fcvm.2022.979581

**Published:** 2022-09-15

**Authors:** Hans Henrik Odland, Torbjørn Holm, Richard Cornelussen, Erik Kongsgård

**Affiliations:** ^1^Department of Cardiology and Pediatric Cardiology, Section for Arrhythmias, Oslo University Hospital, Oslo, Norway; ^2^Department of Cardiology, Section for Arrhythmias, Oslo University Hospital, Oslo, Norway; ^3^Bakken Research Center, Medtronic, Maastricht, Netherlands

**Keywords:** heart failure, cardiac resynchronization therapy, fusion with native conduction, acute hemodynamic response, QRS duration, LV dP/dt_max_

## Abstract

**Background:**

Cardiac resynchronization therapy (CRT) is helpful in selected patients; however, responder rates rarely exceed 70%. Optimization of CRT may therefore benefit a large number of patients. Time-to-peak dP/dt (Td) is a novel marker of myocardial synergy that reflects the degree of myocardial dyssynchrony with the potential to guide and optimize treatment with CRT. Optimal electrical activation is a prerequisite for CRT to be effective. Electrical activation can be altered by changing the electrical wave-front fusion resulting from pacing to optimize resynchronization. We designed this study to understand the acute effects of different electrical wave-front fusion strategies and LV pre-/postexcitation on Td and QRS duration (QRSd). A better understanding of measuring and optimizing resynchronization can help improve the benefits of CRT.

**Methods:**

Td and QRSd were measured in 19 patients undergoing a CRT implantation. Two biventricular pacing groups were compared: pacing the left ventricle (LV) with fusion with intrinsic right ventricular activation (FUSION group) and pacing the LV and right ventricle (RV) at short atrioventricular delay (STANDARD group) to avoid fusion with intrinsic RV activation. A quadripolar LV lead enabled pacing from widely separated electrodes; distal (DIST), proximal (PROX) and both electrodes combined (multipoint pacing, MPP). The LV was stimulated relative in time to RV activation (either RV pace-onset or QRS-onset), with the LV stimulated prior to (PRE), simultaneous with (SIM) or after (POST) RV activation. In addition, we analyzed the interactions of the two groups (FUSION/STANDARD) with three different electrode configurations (DIST, PROX, MPP), each paced with three different degrees of LV pre-/postexcitation (PRE, SIM, POST) in a statistical model.

**Results:**

We found that FUSION provided shorter Td and QRSd than STANDARD, MPP provided shorter Td and QRSd than DIST and PROX, and SIM provided both the shortest QRSd and Td compared to PRE and POST. The interaction analysis revealed that pacing MPP with fusion with intrinsic RV activation simultaneous with the onset of the QRS complex (MPP^*^FUSION^*^SIM) shortened QRSd and Td the most compared to all other modes and configurations. The difference in QRSd and Td from their respective references were significantly correlated (β = 1, R = 0.9, *p* < 0.01).

**Conclusion:**

Pacing modes and electrode configurations designed to optimize electrical wave-front fusion (intrinsic RV activation, LV multipoint pacing and simultaneous RV and LV activation) shorten QRSd and Td the most. As demonstrated in this study, electrical and mechanical measures of resynchronization are highly correlated. Therefore, Td can potentially serve as a marker for CRT optimization.

## Introduction

Cardiac resynchronization therapy (CRT) is helpful in selected patients; however, responder rates rarely exceed 70% (1). Furthermore, CRT may have adverse effects if implemented in the wrong patients (2). Therefore, optimized strategies for implementation of CRT are sought (3). Optimal resynchronization is linked to optimal electrical activation (4). Nevertheless, measures of improved electrical activation do not reflect improvements in cardiac function with CRT in the long term (5), and measures of improved cardiac function do not align with responder rates (6). The left ventricular maximal pressure rise is one marker of cardiac contractility, measured as LV dP/dt_max_. LV dP/dt_max_ is highly dependent on preload and heart rate and could be significantly altered with CRT. We have previously shown how LV dP/dt_max_ is predominantly determined by LV preexcitation and not by resynchronization in patients (7). Therefore, LV dP/dt_max_ is invalid as a biomarker to determine the resynchronization treatment effect. Mechanical recoordination is likely better aligned with long-term response (8–10). Recoordination combines electrical timing and mechanical contraction.

We have recently shown how the time-to-peak left ventricle pressure derivative (Td), a marker of myocardial recoordination, accurately predicts long-term volumetric remodeling in patients in an observational clinical study (11). Td is a measure of the time-delay from the earliest electrical activation until the left ventricular peak pressure rise (dP/dt) or the timing of when the dP/dt_max_ occurs (without considering its amplitude), and therefore combines both electrical activation and resulting mechanical contraction. The peak pressure rise is directly linked to the onset of exponential pressure rise, and this onset marks the time at which the regions of the left ventricle contracts in synergy. Synergistic contractions results in exponential pressure increase. In a typical dyssynchronous heart with left bundle branch block the earliest electromechanical event is the septal beaking (12). The septal beaking is, however, not resulting in exponential pressure rise, but rather in a passive uncoordinated stretch of the adjacent myocardium since the remainder of the left ventricle is resting at this time. Early septal shortening contraction is not coordinated with later lateral wall contraction. The early septal contraction occurs at low loads so that the myofibrils are shortening, mimicking isotonic contraction. Hence, the septal potential energy is wasted at this point.

The exponential increase in pressure occurs later, as electrical wave-front propagation results in more active contraction and subsequent active and passive stiffening of the remaining cardiac walls. Synergistic contraction at this point enables active force generation that results in exponential pressure increase (“isometric state”). The lateral wall performs super-normal work against the maximal load with the delayed pressure increase (13). This mechanism is probably the reason for the delay in peak dP/dt (Td) with dyssynchrony (11). Td shortens once effective biventricular pacing is applied to reflect the reversal of the dyssynchronous mechanisms and to reflect better myocardial coordination. Td has therefore the potential to both diagnose dyssynchrony, but even more so to diagnose effective resynchronization. Effective biventricular pacing should include optimal electrical activation that translate into shortening of Td. In this study, we wanted to investigate how Td and QRS duration responds to single point and multipoint pacing (MPP) with various degrees of optimal and suboptimal electrical fusion in an acute experimental study of patients undergoing CRT implantation.

## Materials and methods

### Ethics statement

This study was an acute-single center observational, experimental hemodynamic study approved by the Regional Committees for Medical and Health Research Ethics in Norway and conducted following the Declaration of Helsinki principles. We obtained written, informed consent from all patients.

### Study population

Heart failure patients admitted for CRT implantation according to current ESC/AHA guidelines were asked to participate in the study. Inclusion criteria were sinus rhythm, New York Heart Association functional class II and III heart failure on optimal medical therapy, QRSd larger than 130 ms and a left ventricular ejection fraction of <35%. In addition, exclusion criteria were age <18 years and above 80 years, ongoing atrial fibrillation and complete atrioventricular block. We successfully positioned the quadripolar LV lead in what we determined was the optimal lateral branch of the coronary sinus in each patient. We considered the optimal coronary sinus branch to be a lateral/ posterolateral branch that allowed positioning of both the distal and the proximal electrodes within the mid to basal portion of the LV wall with adequate pacing capture. An apical and strict anterior position was avoided in all patients. LV pacing (LVP) was set up in an extended bipolar configuration with the cathode on the LV electrode and the anode on the RV defibrillation coil. Therefore, MPP was limited to simultaneous pacing from distal LV electrode to RV coil and proximal LV electrode to RV coil, a configuration superior to other MPP configurations (14). Data from the same patients have been used in a similar study (7).

### Pacing interventions: Pacing mode, electrode configurations and VV-interval (LV pre-/postexcitation)

The atrial pacing (AP) rate was set 10% higher than baseline sinus rhythm, and the AP-QRS interval was measured. First, we paced the right ventricle (RV) at baseline in DDD mode with AV-delay at 80% of the measured AP-QRS interval. Next, we used the AP-QRS interval to calculate the AP-left ventricular paced (LVP) interval to pace the left ventricle (LV) relative to QRS-onset in the fusion with the intrinsic RV activation group (FUSION). Following this, the FUSION group was the only one to allow intrinsic RV activation. In the STANDARD pacing group, the AV-delay to RV pace (RVP) was set to 80% of the AP-QRS interval to avoid intrinsic RV activation and enable standard biventricular pacing (BIVP). We then applied LVP from three different electrode configurations within each intervention group (FUSION/STANDARD). LVP was paced first from the distal electrode (DIST), then from the proximal electrode (PROX), and finally, we combined electrodes DIST+PROX (multipoint pacing, MPP). Also, as in the previously described setup (7), an off-set between LV and RV activation was achieved (VV-interval) as the LV was paced from each electrode configuration with a different extent of LV pre-/postexcitation in three different groups:

LV preexcitation (PRE): LVP between 75 and 25 ms before QRS (FUSION) or RV pace onset (STANDARD).

Simultaneous (SIM): LVP within 25 ms before or after QRS (FUSION) or RV pace onset (STANDARD).

LV postexcitation (POST): LVP between 25 and 75 ms after QRS (FUSION) or RV pace onset (STANDARD).

With this, we created two main groups (FUSION/STANDARD) with three different electrode configurations (DIST, PROX, MPP), each paced repeatedly with three different degrees of LV pre-/postexcitation (PRE, SIM, POST) within each main group in each patient. AV-delay consequently differed between the groups, with AV-delay being shorter in the STANDARD group to avoid intrinsic RV activation compared to the FUSION group. The AV-delay was even shorter when we paced the LV before RV or QRS-onset, as in PRE and SIM. The actual AV-delay within each beat was measured and included in the analyses (the interval from AP to the first ventricular activation; LVP, RVP or QRS-onset). All biventricular pacing interventions were performed similarly in every patient. QRS morphology was visually inspected, compared to successive paced beats and fully paced beats to confirm stable fusion and LV pre-/postexcitation during interventions. We averaged all measurements from 8 to 10 consecutive beats during each pacing intervention.

### Data collection, pacing setup and measurements

We collected electrophysiology signals and ECGs with the BARD Pro EP recording system with Clearsign Amplifier (Boston Scientific Inc.). Pressures were measured *via* femoral artery access from the left ventricle with the Millar Micro-Cath^TM^ pressure sensor catheter (Millar Inc., USA) and collected with the PCU-2000 Pressure Control Unit (Millar Inc., USA). We allowed pressures to stabilize with pacing before measuring the resulting LV dP/dt_max_. Signals were collected in real-time from the recording system to a data acquisition unit (PowerLab, ADInstruments LTD, UK) and analyzed using the LabChart Pro 8.0 software. We performed pacing with the EPS 320 cardiac stimulator (Micropace EP Inc., USA). We determined QRS-onset as the first fluctuation above the isoelectric line, resulting in a complete QRS complex and QRS duration (QRSd) from onset Q to global end of S wave from all ECG leads. Time-to-peak dP/dt (Td) was measured from the earliest of (i) onset of QRS or (ii) onset of the pacing spike until the peak positive first-order derivative of the low-pas (15 Hz) filtered left ventricular pressure curve.

### Statistical analysis

We used linear mixed models (SPSS 26.0) that consider individual baseline values for the repeated measurements. We chose compound symmetry as covariance type for both fixed and random effects, with each subject as random effects, with Bonferroni correction for comparison of main effects. The model with covariates that provided the lowest Akaike's information criteria was selected. The statistical output provides the estimated marginal means ± SEM for each fixed effects group, considering random effects and covariates. It allowed us to analyze the effects of and the interactions between the modes of pacing (FUSION and STANDARD), electrodes used (DIST, PROX, MPP) and pre-/postexcitation (PRE, SIM, POST). We used general linear models to compare groups with no repeated measures. Numbers from descriptive statistics are mean ± SD. A *p*-value of <0.05 was considered statistically significant.

## Results

### Baseline patient characteristics

We included 19 patients with sinus rhythm and a standard indication for a CRT device in the study with characteristics as previously published (7). 84% of the patients had strict LBBB while 16% had intraventricular conduction disease. Demographics are described in Table 1. Table 2 shows the average pre-/postexcitation intervals and AV-delays in the pacing mode groups (FUSION/ STANDARD). In addition, we calculated the onset of QRS to sensed EGM in the LV electrode (Q-LV). Q-LV to distal electrode was 127 ± 19 ms, and Q-LV to proximal electrode was 133 ± 20 ms (mean ± SD), with a linear relationship between the two (β = 0.82, R = 0.86, *P* < 0.01) (7). Table 3 shows the paced intervals within SIM relative to QRS-onset in FUSION/ STANDARD.

**Table 1:** Demographics.

	**All patients (*n* = 19)**
**Age (years)**	64 ± 10 years
**Gender (%)**	
Male	68 (13)
**Weight (kg)**	89 ± 18
**Height (cm)**	176 ± 8
**Heart failure etiology (%)**	
Non-ischemic	53 (10)
Ischemic	42 (8)
Radiation	5 (1)
**Medication (%)**	
ACE inhibitors/ ARB	89 (17)
Beta-blocker	74 (14)
Aldosterone antagonists	53 (10)
Diuretics	47 (9)
**QRS configuration (%)**	
LBBB	84 (16)
IVCD	16 (3)
**QRS duration (ms)**	168 ± 11
**NYHA class**	2.4 ± 0.5
NYHA class II (%)	58 (11)
NYHA class III (%)	42 (8)

LBBB, left bundle branch block; IVCD, intraventricular conduction disease; NYHA class, New York Heart Association class. Numbers are mean ± SD.

**Table 2:** Pacing intervals used for left ventricular pre-/postexcitation and mode of pacing (FUSION/STANDARD).

	**Overall**	**Fusion**	**Standard**
LV preexcitation (PRE)	−47 ± 14 ms	−49 ± 14 ms	−42 ± 13 ms
Simultaneous (SIM)	−2 ± 10 ms	−3 ± 15 ms	−1 ± 7 ms
LV postexcitation (POST)	39 ± 11 ms	40 ± 12 ms	37 ± 10 ms
AV-delay (ms)		199 ± 29 ms	169 ± 36 ms
QRS (ms)	172 ± 12 ms		

LV, left ventricle; AV-delay, atrioventricular paced delay. Numbers are mean ± SD.

**Table 3:** The simultaneous (SIM) pacing group subdivided into within 25 ms before or after RV activation.

	**Fusion**	**Standard**
LV pace 25 ms before	−14 ± 8 ms	−3 ± 6 ms
LV pace 25 ms after	11 ± 7 ms	13 ± 5 ms

Numbers are mean ± SD.

### The effect of mode of pacing (STANDARD vs. FUSION) on QRSd and Td

We analyzed the overall effect of different LV electrodes (DIST, PROX or MPP) used for biventricular stimulation (STANDARD) on QRSd and Td and compared this to LV pacing with fusion with intrinsic RV activation (FUSION). We found a significant difference in QRSd between STANDARD and FUSION (155 ± 2 ms vs. 153 ± 2, *p* < 0.01) and in Td (148 ± 4 ms vs. 145 ± 4 ms, *p* < 0.01). When we included measurements with a VV-interval between −25 and 25 ms only, the difference in QRSd between STANDARD and FUSION was 154 ± 2 ms vs. 144 ± 2 ms (*p* < 0.01), while the difference in Td was 147 ± 4 vs. 136 ± 4 ms (*p* < 0.01). We also analyzed the differences in Td with LV pacing within 25 ms before QRS-onset (FUSION, 141 ± 4 ms) or before RV-pace onset (STANDARD, 146 ± 4 ms, *p* < 0.01) and compared this to LV pacing within 25 ms after QRS-onset (FUSION, 135 ± 4 ms) or before RV-pace onset (STANDARD, 149 ± 4 ms, *p* < 0.01). Similarly, QRSd changed accordingly with LV pacing within 25 ms before QRS with FUSION (151 ± 2 ms) and STANDARD (154 ± 2 ms, *p* < 0.01) pacing and compared this to LV pacing within 25 ms after QRS onset with FUSION (142 ± 2 ms) and STANDARD (155 ± 2 ms, *p* < 0.01) pacing.

### The effect of electrode configuration (DIST vs. PROX vs. MPP) on QRSd and Td

QRSd shortened with MPP compared to both distal and proximal electrodes [152 ± 2 ms (MPP) vs. 157 ± 2 ms (DIST) vs. 155±2 ms (PROX), *p* < 0.01]. Similarly, Td shortened with MPP (144 ± 4 ms, *p* < 0.01) compared to DIST (148 ± 4 ms) and PROX (151 ± 5 ms).

### Interaction between mode of pacing and electrode configuration

**Figure 1:**
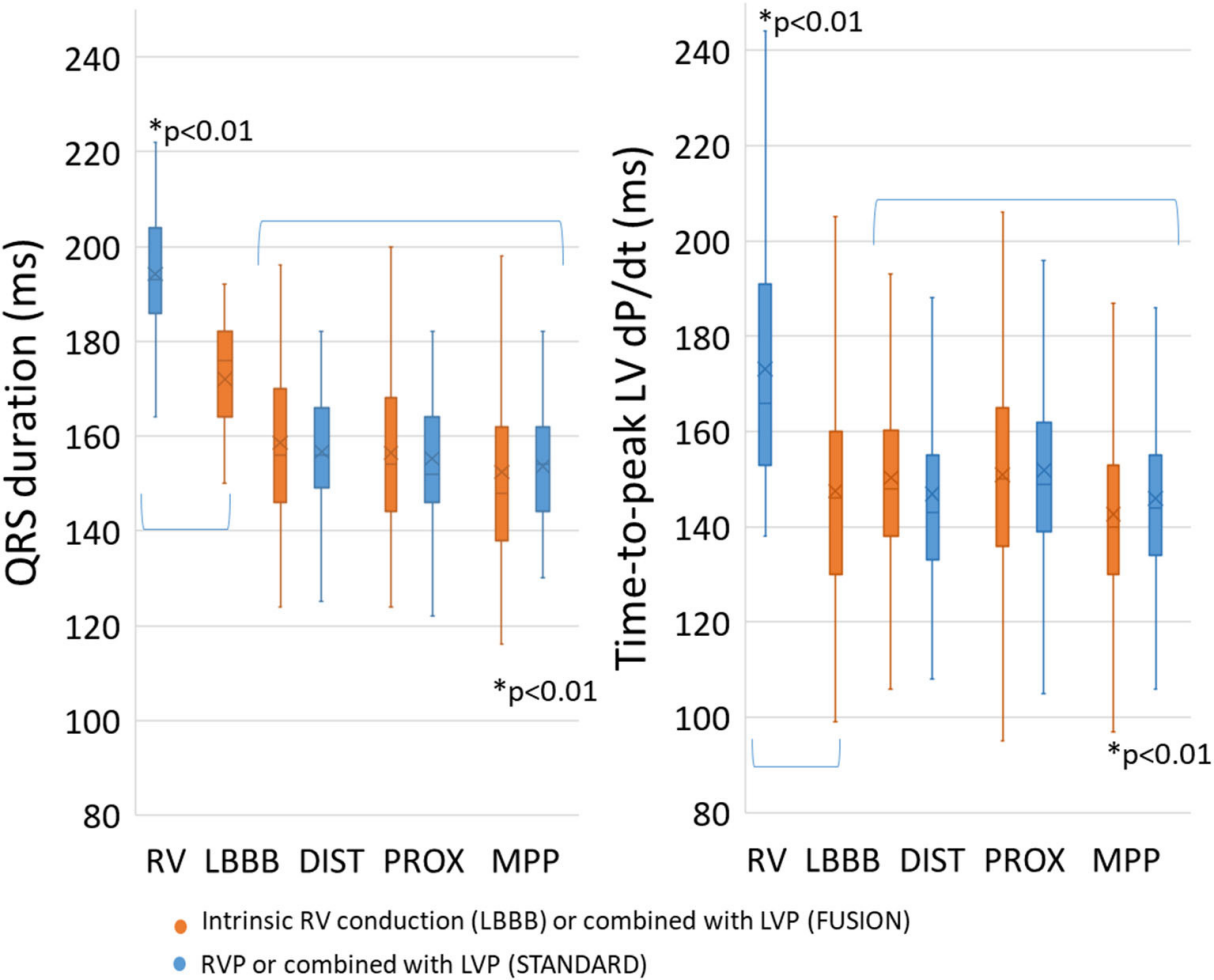
Mode of pacing and fusion with intrinsic RV conduction. A significant increase was found for both QRS duration and time-to-peak dP/dt with RV pacing compared to baseline LBBB. Multipoint pacing with intrinsic RV conduction significantly shortened QRS duration and time-to-peak dP/dt compared to the others (*p* < 0.01). RV, right ventricle; LBBB, left bundle-branch block; DIST, distal electrode; PROX, proximal electrode; MPP, multipoint pacing. * *p* < 0.01 compared to all others.

MPP with fusion with intrinsic conduction (MPP^*^FUSION) provided the shortest QRS and the shortest Td compared to all other measurements (Figure 1). When STANDARD pacing was analyzed separately, we found that MPP shortened Td the most (146 ± 4 ms, *p* < 0.01), followed by DIST (147 ± 4 ms) and PROX (152 ± 4 ms), we also found that MPP shortened QRSd the most (153 ± 2 ms, *p* < 0.01) followed by PROX (155 ± 2 ms) and DIST (157 ± 2 ms). We found a similar pattern for Td and QRSd with FUSION (Td: MPP 141 ± 4 ms vs. DIST 149 ± 4 ms vs. PROX 149 ± 4 ms, *p* < 0.01; QRSd: MPP 150 ± 2 ms vs. PROX 155 ± 2 ms and DIST 156 ± 2 ms and) with significantly lower values for MPP (*p* < 0.01).

### The effect of LV pre-/postexcitation on QRSd and Td

We found that simultaneous pacing (SIM) provided the shortest QRSd (152 ± 2 ms, *p* < 0.01) compared to preexcitation (PRE) at 158 ± 2 ms and postexcitation (POST) at 155 ± 2 ms, with a significant difference also between the latter (*p* < 0.01). We also found a similar pattern for Td; simultaneous pacing (SIM) provided the shortest Td (144 ± 4 ms, *p* < 0.01) compared to preexcitation (PRE) at 153 ± 4 ms and postexcitation (POST) at 148 ± 4 ms, with a significant difference also between the latter (*p* < 0.01).

### The interaction between LV pre-/postexcitation with the mode of pacing

**Figure 2:**
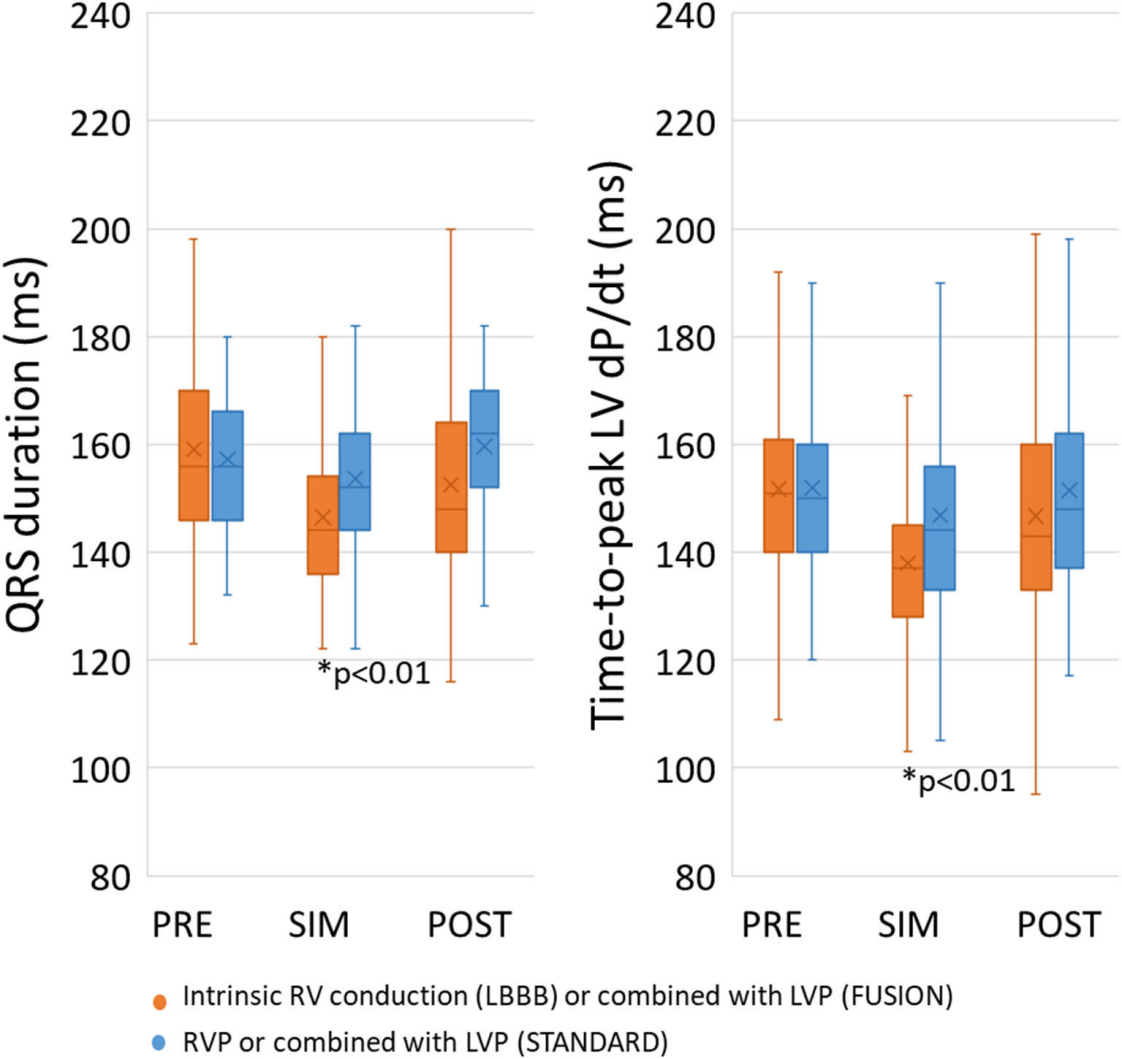
Effect on LV pre-/postexcitation on QRS duration and Td. Simultaneous pacing (SIM) with intrinsic RV conduction shortened both QRS duration and Time-to-peak dP/dt compared to pre-excitation and postexcitation. PRE, pre-excitation of the LV between 75 and 25 ms prior to QRS onset of RV pace onset; SIM, pacing the LV between 25 ms before and 25 ms after QRS onset or RV pace onset; POST, postexcitation of the LV 25–75 ms after QRS onset or RV pace onset. * *p* < 0.01 compared to all others.

Pacing the LV almost simultaneous with QRS onset (SIM) from intrinsic RV activation (FUSION) provided both the shortest QRSd and the shortest Td compared to PRE and POST (Figure 2). We found a weak linear relationship between QRSd and Td (β = 0.24, R = 0.3, *p* < 0.01), QRSd and degree of preexcitation (β = −0.07, R = 0.14, *p* < 0.01) as well as Td and degree of preexcitation (β = −0.07, R = 0.12, *p* < 0.01). When analyzing the effect of pre-/postexcitation on QRSd in the STANDARD group, we found that SIM (154 ± 2 ms, *p* < 0.01) was lower compared to PRE (160 ± 2 ms) and POST (157 ± 2 ms). Similarly, the FUSION group SIM (147 ± 3 ms, *p* < 0.01) was lower compared to PRE (159 ± 3 ms) and POST (153 ± 3 ms). We found similar effects on Td in the STANDARD group, with SIM (146 ± 4 ms, *p* < 0.01) being lower compared to PRE (153 ± 4 ms) and POST (152 ± 4 ms). Td was also lower with SIM (137 ± 4 ms, *p* < 0.01) compared to PRE (153 ± 4 ms) and POST (145 ± 4 ms) in the FUSION group.

### The interaction between mode of pacing, electrode configuration and LV pre-/postexcitation

**Figure 3:**
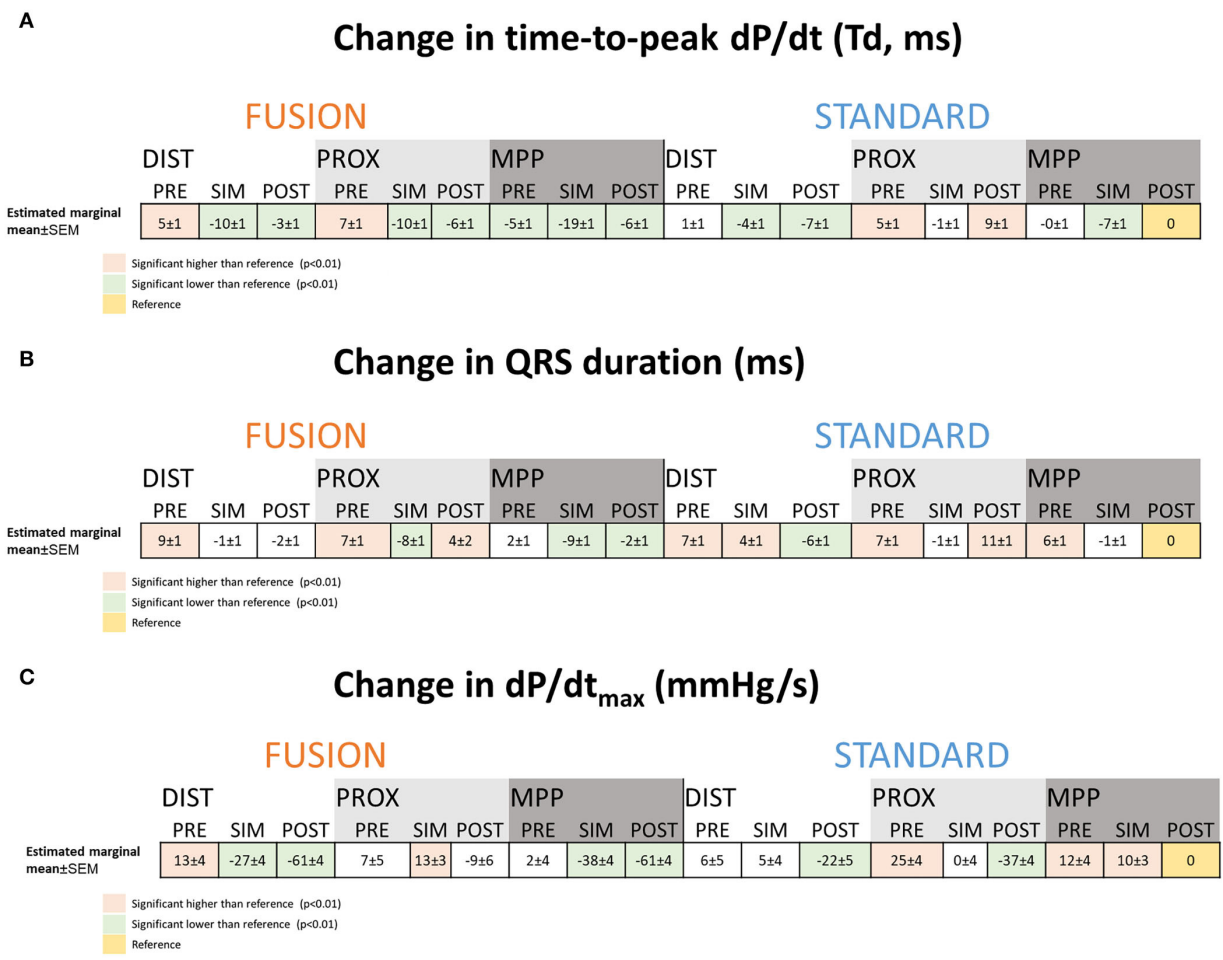
Analysis of the interaction effect between mode of pacing, electrode configuration and LV pre-/postexcitation on the change in Td and QRS duration. The tables show the estimated marginal means and standard error of the linear mixed models analysis for each interaction for time-to-peak dP/dt **(A)**, QRS duration **(B)**, and dP/dt_max_ **(C)**. The numbers are the estimated change from the reference selected as STANDARD*MPP*POST (marked in yellow). Colors that mark the estimates indicate a significant change compared to the reference (*p* < 0.01). Numbers are estimated marginal means ± SEM. DIST, distal electrode; PROX, proximal electrode; MPP, multipoint pacing; PRE—pre-excitation of the LV between 75 and 25 ms prior to QRS onset of RV pace onset; SIM— pacing the LV between 25 ms before and 25 ms after QRS onset or RV pace onset; POST, postexcitation of the LV 25–75 ms after QRS onset or RV pace onset, Td—time-to-peak dP/dt; dP/dt, first order derivative of pressure.

Finally, we analyzed the overall effects of the interaction between pacing mode, electrode position and VV-interval. Figure 3A shows the estimated marginal means. Td shortened with fusion with intrinsic conduction regardless of pre-/postexcitation group and to the most considerable extent with MPP (−19 ± 1 ms, *p* < 0.01) compared to all other interventions. QRSd (Figure 3B) shortened the most with simultaneous pacing (SIM) with FUSION, PROX and MPP (*p* < 0.01), with no difference between the two. We also excluded the measurements with FUSION and pacing within 25 ms after QRS-onset because of a possible bias caused by measuring from QRS onset instead of RV-pace onset and found that simultaneous (SIM) MPP provided the lowest Td and QRSd compared to all other measurements (*p* < 0.01). Figure 3C shows the corresponding change in dP/dt_max_.

### Agreement between electrical and mechanical measures of resynchronization

**Figure 4:**
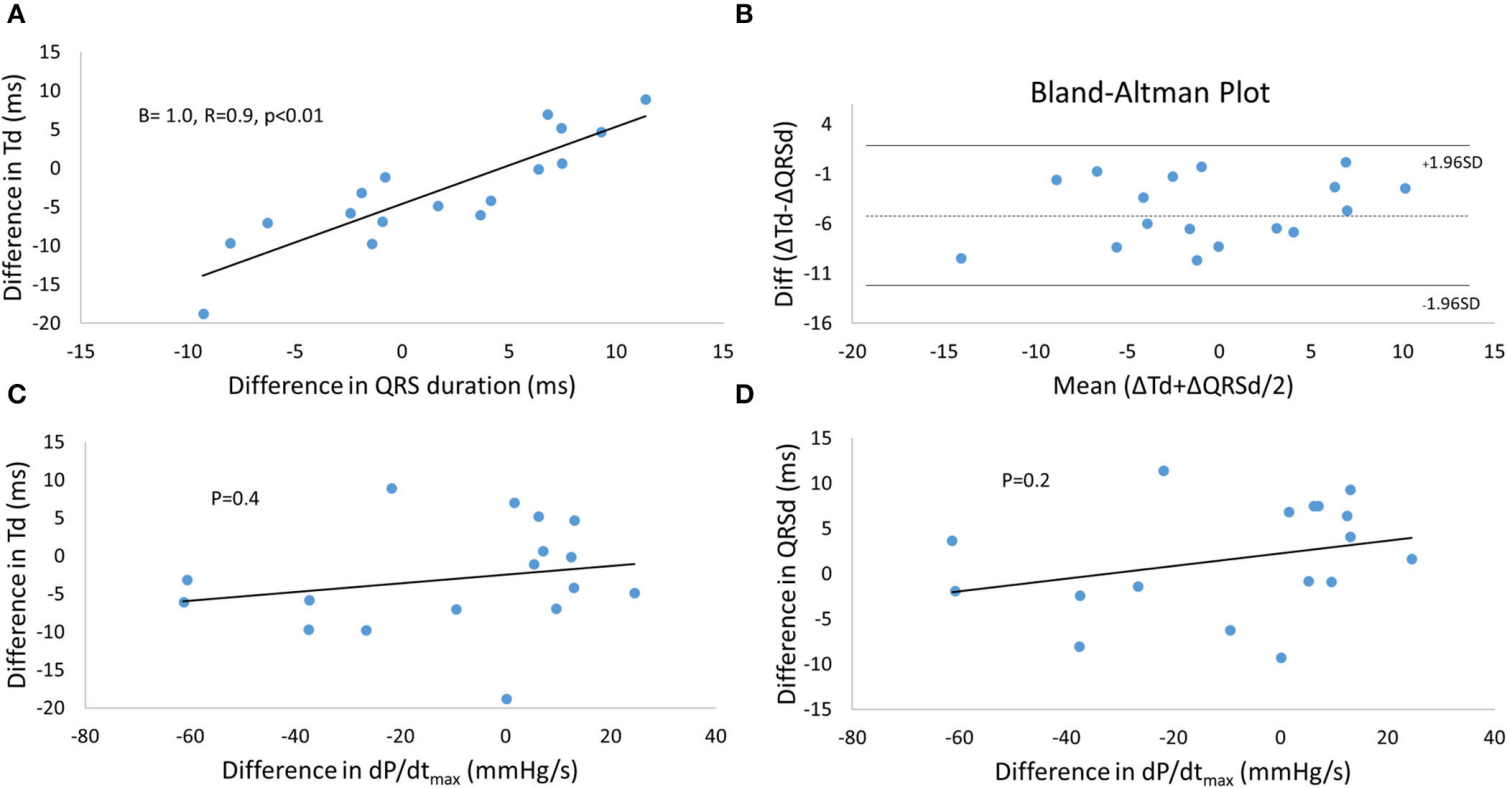
Agreement between mechanical and electrical measures during resynchronization with different modes of pacing and electrode configurations. **(A)** The relationship between the difference in Td and QRSd from the reference (STANDARD*MPP*POST, see Figure 3) as a result of resynchronization, and the Bland-Altman Plot **(B)** to demonstrate the agreement between the measurements. Linear relationships between the difference in Td **(C)** or the difference in QRSd **(D)** and the difference in dP/dt_max_ were not found. Td, time-to-peak dP/dt; dP/dt, first order derivative of pressure.

We used the results from the linear mixed models to display the linear relationship between Td, QRSd and dP/dt_max_ and the corresponding Bland-Altman Plot for the significant linear relationships between the mechanical and electrical measures of resynchronization (Figure 4). The results show that the difference in Td due to different pacing modes, pre-/postexcitation and electrode configuration is similar and linearly related to the difference in QRSd (Figure 4A) with a good agreement and a fixed bias of the mean of −4.6 ms with no further proportional bias (Figure 4B). On the other hand, the difference in dP/dt_max_ was not reflected in either Td or QRSd.

## Discussion

We have previously shown how deformation during the preejection period and synchronicity of the left ventricle are associated with Td. We have also introduced the term synergy to describe mechanical effects from pacing measured as a decrease in Td (11). Shortening Td is associated with more synergy from pacing and a better long-term prognosis (11). In this study, we wanted to demonstrate how stimulation of the LV from two sites at a quadripolar lead (MPP) could be captured by concordant shortening in Td and QRSd, reflecting more synergy and synchrony at the same time. This study is the first to show how MPP improves myocardial synergy on top of electrical synchrony.

**Figure 5:**
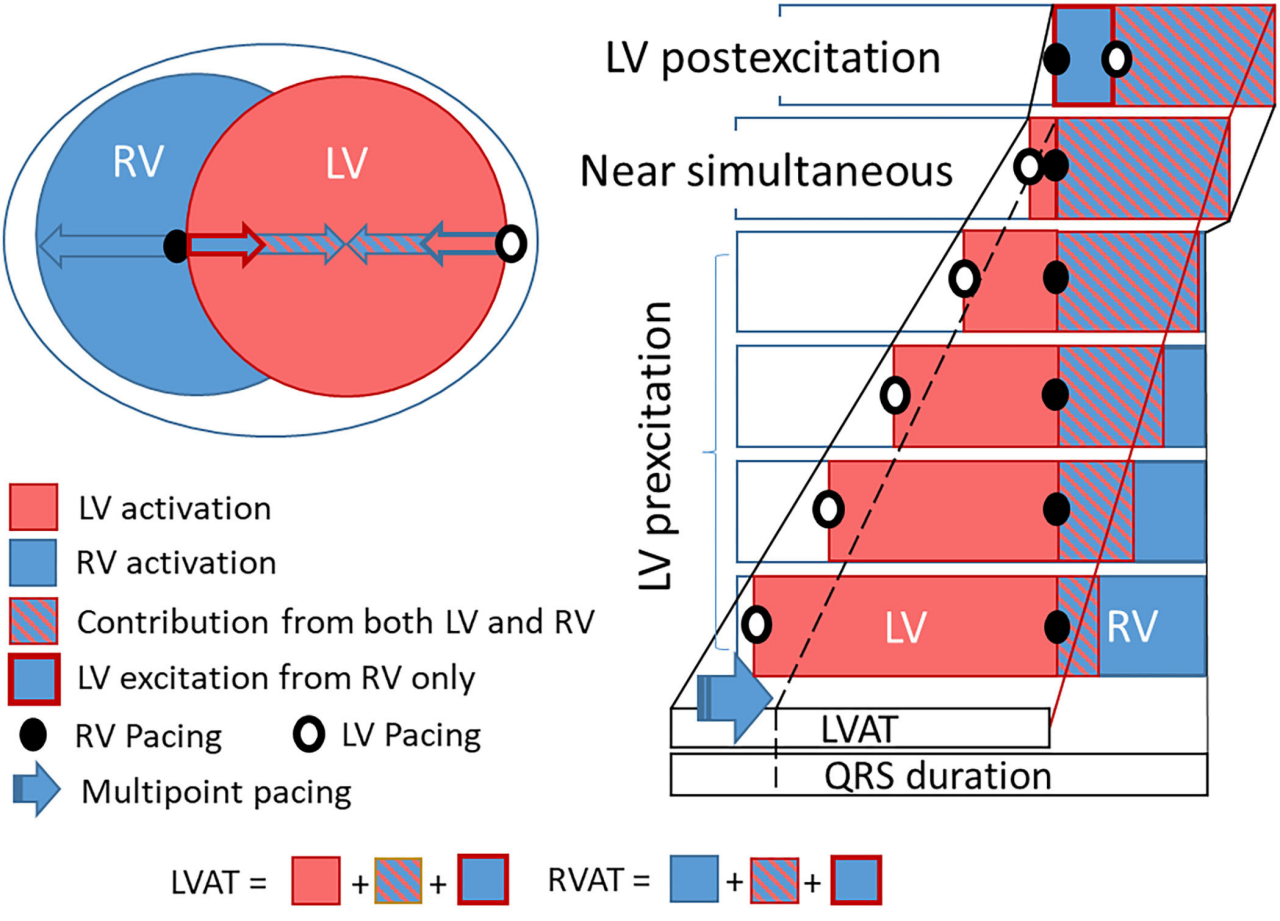
Diagramatic figure showing an ideal electrical activation diagram with the effect of LV pre-/postexcitation and electrodes on LVAT, RVAT and QRS duration. The diagram shows the theoretical change in LVAT and QRS duration relative to LV pre-/postexcitation based on pacing from the LV electrode, with ideally placed RV and LV electrodes. With near maximal LV pre-excitation, the LV is almost fully activated once RV is paced. The residual area, the area not yet activated from the LV as RV is paced, will be recruited from both the RV paced electrode and the LV paced electrode. Hence recruitment in the area will occur in a shorter time. This effect is more pronounced with less LV pre-excitation up to simultaneous pacing when the effect of combined activation of residual areas reaches its maximum, as the residual area recruited from both LV and RV electrodes is at its largest. LVAT and QRS duration shorten with less LV pre-excitation in this diagram down to simultaneous stimulation. Once the LV is postexcitated, the residual area shrinks and LVAT and QRS duration increase (as long as LVAT > RVAT). Shortening LVAT and QRS duration may also occur from multipoint pacing (MPP) with LV pre-excitation (blue arrow and stippled black line). It is clear from this diagram that the LV electrode position may largely affect LVAT and subsequently QRS duration and that intrinsic RV activation (that may shorten both RVAT and LVAT) will impact how this diagram reads. LV, left ventricle; RV, right ventricle; LVAT, left ventricular activation time; RVAT, right ventricular activation time.

RV pacing leads to longer RV activation times compared to intrinsic RV activation and partly explains prolongation in QRSd with RV pacing compared to intrinsic activation, as seen in Figure 1 (15). RV pacing does also affect left ventricular conduction time and contraction patterns (16). The effect of RV pacing on time duration is greater in the LV than in RV (15, 16). Figure 1 shows that Td is lower with intrinsic activation compared to RV pacing showing that a change in electrical activation patterns also translates into changes in contraction patterns. The changes in LV electrical wave-front patterns with RV pacing may increase the LV area of late activation but depends on individual variations and are not necessarily reflected in the QRSd (17). Our study found that MPP shortened QRSd regardless of RV activation patterns (RV pacing or intrinsic RV activation). The effect of MPP on QRSd, therefore, comes on top of the effects seen from RV pacing and indicates that MPP shortens QRSd solely by shortening LVAT (4). We found the same pattern of shortening with MPP. The shortening effect on QRSd and Td is more evident with simultaneous pacing (SIM) than pre- and postexcitation (PRE, POST). The diagrammatic (Figure 5) shows how LV pre-/postexcitation and MPP may affect LVAT, RVAT and QRSd differently. The diagram is an ideal representation of electrode positions in 2D, following the principle of electrical cancellation caused by refractoriness of the myocardial tissue after excitation when two or electrical wave-fronts meet (18). Shortening the LVAT with the recruitment of excitable tissue from two areas is also an effect of multipoint pacing (4, 19).

It is also clear from the illustrative diagram (Figure 5) that changing electrode positions and RV activation times may change the resulting QRSd (20). We do not expect every individual patient have ideal electrode positions, and activation times may vary depending on the underlying myocardial disease. On top of this comes that electrical activation patterns resulting from each electrode may have significant individual variations, and the reaction of the electrical substrate to LV pacing is inconsistent (21). Heart size is another factor that may impact the interpretation of QRSd (22, 23). Although it is clear that QRSd and shortening with CRT are among factors critical for response to CRT (1, 24, 25), QRSd is still an inconsistent and unreliable marker for response (25–28), thus highlighting the need for a measure of resulting mechanical effect (9, 10, 29). The shortening of Td with a shorter QRSd seen in this study is in keeping with animal data showing concordance between synchrony and Td (11).

Another exciting aspect is myocardial discoordination, with some areas shortening while others stretch (10, 12, 13, 30, 31). We have described this as dyssynergistic contraction patterns during the preejection period reflected in time-dependent measures of cardiac contraction, measured as Td (11). The dyssynergistic contraction patterns are contributed to by dyssynchronous electrical activation, typically evident in LBBB or LV pre- and postexcitation. In Figure 5, electrical dyssynchrony of the left ventricle is visualized by areas of LV preactivation resulting from either RV or LV electrodes. Dyssynchrony of the LV is ideally at its minimum with near-simultaneous activation of the electrode pair. Pacing from more electrodes recruits more tissue simultaneously, allowing more synergy from muscular contraction to occur (4).

We found a linear relationship between the change in Td and QRSd, indicating that the two markers reflect a similar underlying substrate. The change was measured relative to the reference being STANDARD^*^MMP^*^POST and is therefore sensitive to any errors in the reference. The correlation plot accounts for this. However, the dependency of the reference may explain the fixed bias in the Bland-Altman plot (Figure 3C). Other factors could also explain the fixed bias between the two measurements, such as the QRSd being dependent on both RV and LV activation while Td only depends on LV activation. Td shortens by a mean of almost 5 ms more than the QRSd. Shortening of LVAT could be partly concealed in the QRSd. Shortening in LVAT resulting from MPP will only translate into a corresponding change in the part of the QRSd solely resulting from LV activation (Figure 5). The portion of the QRSd that results from RV activation only will remain unchanged unless MPP directly affects RV activation (4). Changes in QRSd and Td seen in this study are small, and it is not clear from this study if the effect of MPP on QRSd and Td will translate into better long-term outcomes compared to standard pacing from a single LV electrode. The main point made in the study is that Td is a very sensitive marker of myocardial resynchronization that responds to small changes in electrical activation and wave-front fusion, not necessarily captured by the QRS complex. Td could be valuable for the optimization of resynchronization therapy.

We have previously published data on dP/dt_max_ from the current investigation and shown that dP/dt_max_ is mainly determined by LV preexcitation rather than effective resynchronization (7). As can be seen from Figure 3C, dP/dt_max_ trended to be higher with LV preexcitation and lower with LV postexcitation.

### Clinical implications

QRS morphology and shortening of QRSd with CRT are predictive of response to CRT, however not perfect. Td seems to reflect the mechanical effects of electrical resynchronization directly and could therefore be helpful for optimization of the application of CRT. An increasing amount of evidence points toward restoring LV mechanics as the most critical mechanism for initiating the reverse remodeling processes. Measures of LV mechanical resynchronization are likely more accurate than QRSd for response prediction, as Td directly reflects left ventricular contraction patterns resulting from CRT. In addition, Td shortens with the restoration of LV synergistic contraction patterns following electrical resynchronization. The synergy effect from biventricular pacing measured by Td is predictive of long-term volumetric response. More data is, however, needed to confirm that the shortening of Td following optimization strategies will translate into better patient outcomes.

### Limitations

A small number of patients limits the conclusions drawn from this study. The lack of direct insight into LV activation time intervals and exact electrical propagation in the tissue also limits this study. LV activation time, propagation and activated area over time would be better measures of the effect of fusion and resynchronization than QRSd. MPP may promote better resynchronization in the presence of a scar (19, 32). We used electrodes with limited spacing, and MPP with a longer electrode separation could have provided an even more apparent LV preexcitation effect (33). We did not structure the patients into long-term responders since the numbers of patients were small.

Similarly, we could not stratify patients based on QRS morphology. IVCD patients may have a different Td response to CRT than LBBB patients. We have previously shown that non-responders may increase Td as a response to CRT, and such effects may have impacted our results (11). Another limitation of this paper is the comparison of Td measurements resulting from different references, referenced either from pacing or the onset of QRS. When paced, the measurement of Td and QRSd may be shorter than when measured from the onset of QRS (11). However, when measurements from QRS-onset were excluded, we still found that MPP^*^Sim provided the shortest Td indicating that the effect may not have affected the results. The effects would also be similar for QRSd and Td, and the linear relationship between the changes within the two also supports that the measurement reference may not have significantly impacted the conclusions in this study.

## Conclusion

Multipoint pacing and fusion with right ventricular intrinsic activation improved the electrical resynchronization and the resulting myocardial synergy measured by Td. Pacing the left ventricular electrode(s) simultaneously with the onset of intrinsic right ventricular activation (QRS onset) shortens Td the most in patients with an indication for CRT. The shortening of QRSd was concordant with the shortening in Td. Td has the potential to serve as a marker for CRT optimization.

## Data availability statement

The raw data supporting the conclusions of this article will be made available by the authors, without undue reservation.

## Ethics statement

The studies involving human participants were reviewed and approved by Regional Ethics Committee South-East 2015/1624. The patients/participants provided their written informed consent to participate in this study.

## Author contributions

HO conceived study protocol, organized the study, performed analyses, and wrote the manuscript. TH participated in the study and revised the manuscript. RC conceived study protocol, organized the study, and revised the manuscript. EK conceived study protocol, participated in the study, and revised manuscript. All authors contributed to the article and approved the submitted version.

## Funding

The study was supported by a grant from Helse Sør-Øst RHF.

## Conflict of interest

Author RC was employed by Medtronic. The remaining authors declare that the research was conducted in the absence of any commercial or financial relationships that could be construed as a potential conflict of interest.

## Publisher's note

All claims expressed in this article are solely those of the authors and do not necessarily represent those of their affiliated organizations, or those of the publisher, the editors and the reviewers. Any product that may be evaluated in this article, or claim that may be made by its manufacturer, is not guaranteed or endorsed by the publisher.

## References

[1] Okafor O Zegard A van Dam P Stegemann B Qiu T Marshall H. Changes in QRS area and QRS duration after cardiac resynchronization therapy predict cardiac mortality, heart failure hospitalizations, and ventricular arrhythmias. J Am Heart Assoc. (2019) 8:e013539. 10.1161/jaha.119.013539 31657269 PMC6898809

[2] Stankovic I Stefanovic M Prinz C Ciarka A Daraban AM Kotrc M. The association of mechanical dyssynchrony and resynchronization therapy with survival in heart failure with a wide QRS complex: a two-world study. Int J Cardiovasc Imaging. (2020) 36:1507–14. 10.1007/s10554-020-01865-x 32356183

[3] Mullens W Auricchio A Martens P Witte K Cowie MR Delgado V. Optimized Implementation of cardiac resynchronization therapy—a call for action for referral and optimization of care. Eur J Heart Fail. (2020) 22:2349–69. 10.1002/ejhf.2046 33136300

[4] Albatat M Arevalo H Bergsland J Strom V Balasingham I Odland HH. Optimal pacing sites in cardiac resynchronization by left ventricular activation front analysis. Comput Biol Med. (2021) 128:104159. 10.1016/j.compbiomed.2020.104159 33301952

[5] Stephansen C Sommer A Kronborg MB Jensen JM Nørgaard BL Gerdes C. Electrically vs. imaging-guided left ventricular lead placement in cardiac resynchronization therapy: a randomized controlled trial. EP Europace. (2019) 21:1369–77. 10.1093/europace/euz184 31274152

[6] Manav Sohal M Shoaib Hamid M Giovanni Perego M Paolo Della Bella M Shaumik Adhya M John Paisey M. A multicenter prospective randomized controlled trial of cardiac resynchronization therapy guided by invasive dP/dt. Heart Rhythm O2. (2021) 2:19–27. 10.1016/j.hroo.2021.01.005 34113901 PMC8183864

[7] Odland HH Holm T Gammelsrud LO Cornelussen R Kongsgaard E. Determinants of LV dP/dt. Open Heart. (2021) 8:1615. 10.1136/openhrt-2021-001615 33963078 PMC8108692

[8] Aalen JM Remme EW Larsen CK Andersen OS Krogh M Duchenne J. Mechanism of abnormal septal motion in left bundle branch block: role of left ventricular wall interactions and myocardial scar. JACC Cardiovasc Imaging. (2019) 12:2402–13. 10.1016/j.jcmg.2018.11.030 30772230

[9] Duchenne J Aalen JM Cvijic M Larsen CK Galli E Bezy S. Acute redistribution of regional left ventricular work by cardiac resynchronization therapy determines long-term remodelling. Eur Heart J Cardiovasc Imaging. (2020) 21:619–28. 10.1093/ehjci/jeaa003 32031587

[10] Aalen JM Donal E Larsen CK Duchenne J Lederlin M Cvijic M. Imaging predictors of response to cardiac resynchronization therapy: left ventricular work asymmetry by echocardiography and septal viability by cardiac magnetic resonance. Eur Heart J. (2020) 41:3813–23. 10.1093/eurheartj/ehaa603 32918449 PMC7599033

[11] Odland HH Villegas-Martinez M Ross S Holm T Cornelussen R Remme EW. Shortening of time-to-peak left ventricular pressure rise (Td) in cardiac resynchronization therapy. ESC Heart Fail. (2021) 8:5222–36. 10.1002/ehf2.13601 34514746 PMC8712829

[12] Remme EW Niederer S Gjesdal O Russell K Hyde ER Smith N. Factors determining the magnitude of the pre-ejection leftward septal motion in left bundle branch block. Europace. (2016) 18:1905–13. 10.1093/europace/euv381 26612883 PMC5291191

[13] Russell K Eriksen M Aaberge L Wilhelmsen N Skulstad H Remme EW. A novel clinical method for quantification of regional left ventricular pressure-strain loop area: a non-invasive index of myocardial work. Eur Heart J. (2012) 33:724–33. 10.1093/eurheartj/ehs016 22315346 PMC3303715

[14] Thibault B Dubuc M Khairy P Guerra PG Macle L Rivard L. Acute haemodynamic comparison of multisite and biventricular pacing with a quadripolar left ventricular lead. Europace. (2013) 15:984–91. 10.1093/europace/eus435 23447571

[15] Varma N Jia P Ramanathan C Rudy Y. RV electrical activation in heart failure during right, left, and biventricular pacing. JACC Cardiovasc Imaging. (2010) 3:567–75. 10.1016/j.jcmg.2009.12.017 20541711 PMC2976709

[16] O'Donnell D Manyam H Pappone C Park SJ Leclercq C Lunati M. Ventricular activation patterns during intrinsic conduction and right ventricular pacing in cardiac resynchronization therapy patients. Pacing Clin Electrophysiol. (2021) 44:1663–70. 10.1111/pace.14329 34319603

[17] Varma N. Left ventricular electrical activation during right ventricular pacing in heart failure patients with LBBB: visualization by electrocardiographic imaging and implications for cardiac resynchronization therapy. J Electrocardiol. (2015) 48:53–61. 10.1016/j.jelectrocard.2014.09.002 25301520

[18] Bank AJ Gage RM Schaefer AE Burns KV Brown CD. Electrical wavefront fusion in heart failure patients with left bundle branch block and cardiac resynchronization therapy: implications for optimization. J Electrocardiol. (2020) 61:47–56. 10.1016/j.jelectrocard.2020.05.015 32526538

[19] Albatat M Bergsland J Arevalo H Odland HH Wall S Sundnes J. Multisite pacing and myocardial scars: a computational study. Comput Methods Biomech Biomed Eng. (2020) 23:248–60. 10.1080/10255842.2020.1711885 31958019

[20] Derval N Bordachar P Lim HS Sacher F Ploux S Laborderie J. Impact of pacing site on QRS duration and its relationship to hemodynamic response in cardiac resynchronization therapy for congestive heart failure. J Cardiovasc Electrophysiol. (2014) 25:1012–20. 10.1111/jce.12464 24891271

[21] Varma N. Variegated left ventricular electrical activation in response to a novel quadripolar electrode: visualization by non-invasive electrocardiographic imaging. J Electrocardiol. (2014) 47:66–74. 10.1016/j.jelectrocard.2013.09.001 24099886

[22] Rickard J Baranowski B Grimm RA Niebauer M Varma N Tang WHW. Left ventricular size does not modify the effect of QRS duration in predicting response to cardiac resynchronization therapy. Pacing Clin Electrophysiol. (2017) 40:482–7. 10.1111/pace.13043 28164328

[23] Varma N Lappe J He J Niebauer M Manne M Tchou P. Sex-specific response to cardiac resynchronization therapy: effect of left ventricular size and QRS duration in left bundle branch block. JACC Clin Electrophysiol. (2017) 3:844–53. 10.1016/j.jacep.2017.02.021 29759781

[24] Korantzopoulos P Zhang Z Li G Fragakis N Liu T. Meta-analysis of the usefulness of change in qrs width to predict response to cardiac resynchronization therapy. Am J Cardiol. (2016) 118:1368–73. 10.1016/j.amjcard.2016.07.070 27634027

[25] Molhoek SG Vane L Bootsma M Steendijk P Van Der Wall EE Schalij MJ. QRS duration and shortening to predict clinical response to cardiac resynchronization therapy in patients with end-stage heart failure. Pacing Clin Electrophysiol PACE. (2004) 27:308–13. 10.1111/j.1540-8159.2004.00433.x 15009855

[26] Biton Y Kutyifa V Cygankiewicz I Goldenberg I Klein H McNitt S. Relation of QRS duration to clinical benefit of cardiac resynchronization therapy in mild heart failure patients without left bundle branch block: the multicenter automatic defibrillator implantation trial with cardiac resynchronization therapy substudy. Circ Heart Fail. (2016) 9:e002667. 10.1161/circheartfailure.115.002667 26823498

[27] Engels EB Mafi-Rad M van Stipdonk AM Vernooy K Prinzen FW. Why QRS duration should be replaced by better measures of electrical activation to improve patient selection for cardiac resynchronization therapy. J Cardiovasc Transl Res. (2016) 9:257–65. 10.1007/s12265-016-9693-1 27230674 PMC4990608

[28] Gijsberts CM Benson L Dahlström U Sim D Yeo DP Ong HY. Ethnic differences in the association of QRS duration with ejection fraction and outcome in heart failure. Heart. (2016) 102:1464–71. 10.1136/heartjnl-2015-309212 27402805 PMC5013108

[29] Ross S Nestaas E Kongsgaard E Odland HH Haland TF Hopp E. Septal contraction predicts acute haemodynamic improvement and paced QRS width reduction in cardiac resynchronization therapy. Eur Heart J Cardiovasc Imaging. (2020) 21:845–52. 10.1093/ehjci/jez315 31925420

[30] Wouters PC Leenders GE Cramer MJ Meine M Prinzen FW Doevendans PA. Acute recoordination rather than functional hemodynamic improvement determines reverse remodelling by cardiac resynchronisation therapy. Int J Cardiovasc Imaging. (2021) 37:1903–11. 10.1007/s10554-021-02174-7 33547623 PMC8255256

[31] Gjesdal O Remme EW Opdahl A Skulstad H Russell K Kongsgaard E. Mechanisms of abnormal systolic motion of the interventricular septum during left bundle-branch block. Circ Cardiovasc Imaging. (2011) 4:264–73. 10.1161/circimaging.110.961417 21393502

[32] Ginks MR Duckett SG Kapetanakis S Bostock J Hamid S Shetty A. Multi-site left ventricular pacing as a potential treatment for patients with postero-lateral scar: insights from cardiac magnetic resonance imaging and invasive haemodynamic assessment. Europace. (2012) 14:373–9. 10.1093/europace/eur336 22045930

[33] Heckman LIB Kuiper M Anselme F Ziglio F Shan N Jung M. Evaluating multisite pacing strategies in cardiac resynchronization therapy in the preclinical setting. Heart Rhythm O2. (2020) 1:111–9. 10.1016/j.hroo.2020.03.003 34113865 PMC8183878

